# The role of grit and resilience in children with reading disorder: a longitudinal cohort study

**DOI:** 10.1007/s11881-021-00238-w

**Published:** 2021-07-29

**Authors:** Bushra Hossain, Yingtong Chen, Stephen Bent, China Parenteau, Felicia Widjaja, Stephanie L. Haft, Fumiko Hoeft, Robert L. Hendren

**Affiliations:** 1 Department of Psychiatry, University of California, San Francisco, 401 Parnassus Ave, San Francisco, CA 94143 USA; 2 Department of Epidemiology and Biostatistics, University of California, San Francisco, CA, USA; 3 Department of Psychology, University of California, Berkeley, CA, USA; 4 Department of Psychological Sciences, University of Connecticut, Mansfield, CT, USA

**Keywords:** Academic performance, Anxiety, Depression, Dyslexia, Grit, Mental health, Quality of life, Reading disorder, Resilience

## Abstract

Prior studies have suggested that grit and resilience predict both academic and career success. However, these qualities have not been examined in children with reading disorder (RD). We therefore investigated whether grit and resilience were associated with anxiety, depression, academic performance, and quality of life (QOL) in these students. This 3-year longitudinal cohort study included 163 participants with RD from 3 schools. Evaluations were completed by parents and/or teachers every 3 months. The Grit and Resilience Scale was adapted from the 10-item Connor-Davidson Resilience Scale and the 12-item Grit Scale. Outcome measures included anxiety (School Anxiety Scale – Teacher Report and the 8-item Spence Children’s Anxiety Scale), depression (Short Mood and Feelings Questionnaire), academic performance, and QOL (Pediatric QOL Inventory 4.0). Multivariate linear regression models (adjusting for age and sex) assessed the associations at baseline. Repeated measures analysis using mixed-effects models assessed the relationship longitudinally. There were statistically significant associations between grit and resilience and all outcomes at baseline and over time. After adjusting for age and sex, improved grit and resilience was associated with decreased anxiety (β =  − 0.4, *p* < 0.001) and improved academic performance (β = 0.5, *p* < 0.001) when grit and resilience was measured by teachers, as well as decreased depression (β =  − 0.3, *p* < 0.001) and improved QOL (β = 0.6, *p* < 0.001) when grit and resilience was measured by parents. Grit and resilience are significantly related to mental health, academic performance, and QOL in children with RD. This suggests that interventions to improve grit and resilience may lead to positive benefits.

**Supplementary Information:**

The online version contains supplementary material available at 10.1007/s11881-021-00238-w.

The term “grit” was defined as “perseverance and passion for long term goals” in a widely quoted 2007 study by Duckworth et al. (2007). This landmark paper built upon comments and research dating back to the late 1800s suggested that ability alone did not predict success, but that “zeal and a capacity for hard labor” were essential components. The authors developed a novel Grit Scale, which embedded grit in two main facets (perseverance of effort and consistency of interest), and tested the predictive ability in several studies, finding that higher grit was associated with higher grade point average (GPA) among Ivy League students, retention among military recruits, and ranking in a national spelling bee (Duckworth et al., 2007). Subsequent studies have confirmed that grit is associated with students’ current and future GPA in high schools (Duckworth & Quinn, 2009; Muenks et al., 2017a, b). Grit was also found to predict career success and especially career engagement, even after controlling for cognitive ability and sociodemographic characteristics (Lechner et al., 2019).

Resilience, on the other hand, focuses on the “dynamic process that enables positive adapting to stressors,” or the ability to “bounce back” after adversity or failure (Straus et al., 2020). Although there has been much variation on how resilience is operationalized, most researchers describe resilience as being multifactorial and dynamic, and involving the return to a positive mental state after adversity by using a collection of internal and external protective factors and resources, such as personal strengths, good social skills, strong attachment to family, and social support (Dray et al., 2017). Indeed, resilience involves a dynamic interplay between individual, environmental, and sociocultural factors (Lavin Venegas et al., 2019) and has been positively associated with positive indicators of mental health (Hu et al., 2015).

There has been much research on how grit and/or resilience is related to mental health, academic achievement, and quality of life. Increased levels of resilience have been associated with decreased symptoms of both anxiety (Conway & McDonough, 2006; Worku et al., 2019) and depression (Askeland et al., 2015; Conway & McDonough, 2006; Hjemdal et al., 2007) in children and adolescents in a number of different studies. Grit has been shown to have a negative correlation with depression among adolescents (Datu et al., 2018) and with both anxiety and depression among young adults (Musumari et al., 2018; Zhang et al., 2018). Resilience has been positively associated with health-related quality of life in a number of patient populations, including cancer patients (Popa-Velea et al., 2017; Zhang et al., 2017), adolescents with chronic health problems (Kim et al., 2019), and individuals with eating disorders (Calvete et al., 2018), while grit has been positively associated with health-related quality of life among college students (Sharkey et al., 2017). The role of grit on academic performance has been more controversial. While many studies found a positive impact of grit on academic performance, recent reviews have indicated that the relationship may be weaker and more complex than previously thought. A recent meta-analysis (Credé et al., 2017) and systematic review (Christopoulou et al., 2018) found such associations to be weak to moderate at best. Rather, some argue that the perseverance of effort facet of grit is a stronger predictor of academic performance compared to the consistency of interest facet and even compared to grit in general (Christopoulou et al., 2018; Credé et al., 2017; Katherine Muenks et al., 2017a, b).

It should be noted that although almost all the previous studies were cross-sectional in nature (including those reported in the meta-analysis and systematic review for academic outcomes), all except one study (Worku et al., 2019) considered grit or resilience to be the predictor variable in their analyses, whereas other variables including anxiety, depression, quality of life, or academic performance were defined as the outcomes. In a longitudinal study among adolescents, the perseverance facet of grit in eighth grade was found to predict school achievement (GPA) and engagement in ninth grade (Tang et al., 2019). In a study among nursing students, resilience was found to have a significant longitudinal effect on psychological well-being (after 18 months); however, there was no evidence for an inverse relationship, i.e., psychological well-being did not predict resilience (Ríos-Risquez et al., 2018). In contrast, longitudinal reciprocal relationships between resilience and quality of life were found in a cohort of adults with eating disorder (Calvete et al., 2018). Considering that there are so few longitudinal studies examining grit and/or resilience, this is an area that warrants further research.

Some have noted that resilience is, by definition, an essential component of the persistence of effort aspect of grit (Stoffel & Cain, 2018). Measuring grit and resilience is complex, but it is nonetheless clear that these “non-cognitive” constructs are strong predictors of mental health, quality of life, and academic and career success. Given the importance of grit and resilience to attaining success, it seems crucial to examine whether they are also predictive and helpful in adolescents with specific challenges, including those with reading disorder (RD), such as dyslexia, which is a decoding-based reading disability.

Approximately 7% of all children have RD (Peterson & Pennington, 2015), which can create numerous challenges that generally present as children enter their academic phase of life. Children with RD have higher rates of academic failure, are 2.5 times more likely to drop out of high school (Haft et al., 2016), and also have higher rates of anxiety, depression, and conduct problems (Cederlof et al., 2017). Since children with RD have such significant challenges to overcome, it seems especially important to examine grit and resilience in this population to determine if higher ratings on these constructs are associated with positive outcomes (such as low ratings of anxiety/depression symptomology and general academic performance) and therefore could be a target of intervention. Furthermore, given the contradictory evidence of grit’s effect on academic performance among the typically developing population, investigating this relationship in the RD population, who face significantly greater academic challenges, seems worthwhile.

To our knowledge, there have been very few studies that have examined the predictive ability of grit and resilience on outcomes in children with RD. One study examined the impact of a “school-based resilience program” on outcomes in 23 students with dyslexia compared to 79 students without dyslexia (Firth et al., 2013). Students in the treatment program had improvements in “locus of control” and “nonproductive coping,” but the study did not report changes in academic outcomes or other measures of overall health and quality of life.

We therefore conducted a prospective cohort study in three schools specializing in the treatment of children with RD. Our initial objective was to develop a combined measure of grit and resilience that would be appropriate for the RD population. Our primary objective was to determine whether this new grit and resilience measure was associated with academic performance, mental health (anxiety and depression), and overall quality of life, both at baseline and over time in children with RD. We also had an exploratory aim to see whether grit and resilience itself changed over time on average in this cohort.

We sought to create a new scale for measuring grit and resilience upon advice from the schools’ teachers who felt that many items in the existing scales measuring grit and resilience separately were not relevant to the RD population (e.g., Resilience in Illness Scale) or were not age-appropriate for this sample (e.g., Resilience Scale for Adolescents). Our new Grit and Resilience Scale borrowed items from two validated scales for grit (Duckworth et al., 2007) and resilience (Connor & Davidson, 2003), respectively, both of which (or their shorter/longer versions) were used in many of the studies discussed above. The two constructs were combined because some suggest resilience to be an inherent attribute of grit (Stoffel & Cain, 2018), and to reduce survey completion burden on the respondents by allowing them to complete one survey rather than two.

Although previous studies used self-reports to measure grit, resilience, and all our outcomes of interest, we decided to use parent and teacher reports for all measures in this study, as we believed that to be more suitable for our cohort of children with RD. As children with RD face difficulty with reading and writing, we felt that it could be too difficult for them (especially the younger participants; some aged 6 years) to complete self-reports of multiple surveys at regular intervals throughout the study. In contrast, the previous studies using self-reported measures had consisted of typically developing children and older age groups (e.g., adolescents and adults) who were more capable of understanding and accurately completing these scales. We also hoped that the parent and teacher reports would shed light on whether the grit and resilience a child displayed varied depending on the environment (home vs. school) and would also reduce social desirability bias that is often seen as a drawback of self-reports.

Therefore, the primary purpose of this cohort study was to investigate whether parent and teacher reports of our newly developed grit and resilience scale were associated with anxiety, depression, academic performance, and quality of life, both at baseline and over time, in children with RD.

## Methods

### Protocol

The study was approved by the University of California San Francisco (UCSF) Committee on Human Research on 09/28/2016. Questionnaires measuring grit and resilience and other outcome factors (anxiety, depression, academic performance, and quality of life) were filled out by parents and teachers approximately every 3 months in an academic school year, up to ten times. Data was collected through an online secure research platform called eBit (evidence-based intervention and treatment) on the following dates: April 2017, June 2017, October 2017, January 2018, May 2018, November 2018, April 2019, June 2019, October 2019, and February 2020.

### Participants and consent

One hundred and sixty-three participants (aged between 6 and 16 years) were recruited by email from three specialized education schools for children with RD in the San Francisco Bay Area, CA. All participants were required to have RD to be enrolled in the schools and the study. The local schools were all recognized as schools that specialize in the education of children with dyslexia and other forms of RD. Recruitment for participants was ongoing throughout the study period, resulting in staggered entry over the 3 years. This led to differential follow-up times for the participants, with the earliest participants having up to ten timepoints of measurement (Supplementary Table 1). Informed consent was obtained from parents before any research activities were started.

### Measures

#### Grit and resilience

The primary predictor for this study was measured by the Grit and Resilience Scale, which measures how well a child adapts to challenging or difficult situations. It was adapted from the 10-item Connor-Davidson Resilience Scale (CD-RISC) (Campbell-Sills & Stein, 2007) and the 12-item Grit Scale (Duckworth et al., 2007). The Grit and Resilience Scale has both parent and teacher versions.

The UCSF research team met with the teachers and leadership at the schools to review the individual grit and resilience scales. However, none of the available scales seemed to completely capture what teachers found most relevant for dyslexia and other forms of RD. In addition, they surmised that using more than one scale would be too long for the teachers and parents to complete multiple times over the study period. Therefore, the UCSF team took the items deemed most relevant from both scales and created the modified Grit and Resilience Scale to increase survey completion rate. In many cases, they also changed the wording to better adapt to the RD population.

Five items in the new Grit & Resilience Scale were adapted from the 12-item Grit Scale. In all cases, the new items were thematically similar to the ones that they were inspired from, rather than identical copies. In addition, they were worded in a way to reflect the third-person rating (rather than self-report) and some items were reverse-worded to maintain scoring consistency (i.e., higher is better for all items). For instance, item #8 from the Grit Scale “I have difficulty maintaining my focus on projects that take more than a few months to complete” was changed to “Uses strategies and tools to regain or maintain focus,” or item #9 from Grit Scale “I finish whatever I begin” was adapted as “Takes action to complete challenging tasks.”

Seven items in the new Grit & Resilience Scale were adapted from the 10-item CD-RISC. Among these, 4 items were worded almost identically, with the minor changes reflecting the change in rater. For instance, item #1 from CD-RISC 10 “I am able to adapt when changes occur” became “Adapts when changes occur.” The other 3 items were thematically similar and were therefore worded differently. For instance, item #9 from 10-item CD-RISC “I think of myself as a strong person…” was reflected in “Expresses positive self-perception” on the new scale.

The parent version of the scale contained 17 questions (answered on a 5-point Likert scale), with a possible final score range of 0–68. Among these, 5 questions were from the Grit Scale, 7 questions from CD-RISC (with 2 questions with overlapping themes that therefore were not duplicated), and 7 additional questions from the teachers. On the other hand, the teacher version contained 21 questions (5-point Likert scale), with a possible final score range of 0–84. Among these, 5 questions were from the Grit Scale, 7 questions from CD-RISC (with 2 questions with overlapping themes that therefore were not duplicated), and 11 additional questions. A higher score indicated better grit and resilience. Both versions of the scale are reported in the Appendixes 1 and 2.

The Grit & Resilience Scale was tested for its psychometric properties. It showed excellent internal consistency at baseline, with the parent-reported scale having a Cronbach’s alpha of 0.91 and the teacher-reported scale having an alpha of 0.94. It also showed good test–retest reliability, with the parent version having an intraclass correlation (ICC) of 0.72 (95% CI: 0.65 to 0.78) and the teacher version having an ICC of 0.75 (95% CI: 0.70 to 0.80). We tested for convergent validity using the total difficulties score of the Strengths and Difficulties Questionnaire (SDQ), which has been used to measure resilience in a number of cohort studies (King et al., 2021; Young et al., 2019) and to test for convergent validity in another study evaluating a novel resilience scale (Suzuki et al., 2015). Although SDQ itself does not operationally define resilience, the studies that used it to measure resilience defined the construct as follows: “positive adaptation or better-than-expected outcomes in the context of…adversity” (King et al., 2021), “[showing] positive outcomes despite the presence of adversity” (Young et al., 2019) and “the process of positive adaptation to…difficulties” (Suzuki et al., 2015), all of which were very similar to our own definition of resilience in this study: “the ability to bounce back after adversity or failure.” The Grit & Resilience Scale achieved convergent validity with SDQ, with parent-reported measures having a correlation coefficient (r) of − 0.74 (*p* < 0.001) and teacher-reported measures having an r of − 0.73 (*p* < 0.001). SDQ was obtained for all participants at all timepoints. We tested for divergent validity using the Kiddie Sluggish Cognitive Tempo Rating Scale (K-SCT) (McBurnett et al., 2014), which measures symptoms of Sluggish Cognitive Tempo (SCT), including daydreaming, lethargy, drowsiness, lack of motivation, and mental confusion (Sevincok et al., 2020). To our knowledge, SCT has not been associated with grit and resilience in prior studies, and the operational definitions of the constructs indicate SCT to be completely unrelated to grit and resilience as well, which is why we used K-SCT for measuring divergent validity. After correcting for attenuation due to measurement error, we found weak correlations with K-SCT (parent-reported: r =  − 0.41, *p* < 0.001; teacher-reported: r =  − 0.50, *p* < 0.001). However, the low magnitude of the correlation coefficients and the fact they are significantly lower than the convergent validity correlations (parent-reported: z =  − 6.57, *p* < 0.0001; teacher-reported: z =  − 6.56, *p* < 0.0001) suggest that the Grit & Resilience Scale measures unique socioemotional traits that is different from SCT. K-SCT was introduced to the study in April 2019 and was measured for all existing participants from that point forth.

Factor analysis revealed that the Grit and Resilience Scale loaded as three factors for the parent-reported version and as four factors for the teacher-reported version, with the items taken from the Grit Scale loading as one group, the items taken from the CD-RISC loading as another group, and the additional items loading as the third group and fourth group (fourth for teacher-reported only). Considering that the Grit and Resilience Scale measures several related but somewhat distinct concepts (such as resistance of effort, consistency of interest, ability to bounce back after adversity, or self-advocacy), this was not surprising. Nevertheless, we opted to use total scores in all subsequent analyses due to the relatedness of these concepts (Stoffel & Cain, 2018) and the excellent internal consistency (as indicated by the high Cronbach’s alpha scores) for both parent and teacher versions of the full scale. In order to confirm that the high Cronbach’s alpha scores were not a result of the larger number of items in the full scale but rather a true measure of reliability, we calculated the alpha for each individual factor as well (Tavakol & Dennick, 2011), and still found them to demonstrate good to excellent internal consistency (Cronbach’s alpha: 0.81–0.90). In addition, many previously published and validated scales with multiple factors also derive and use total scale scores. For instance, the CD-RISC (Connor & Davidson, 2003) and the Pediatric Quality of Life Inventory 4.0 (Varni et al., 2001) both yielded five factors, and the 12-item Grit Scale had two factors (Duckworth et al., 2007), but they all had good reliability and generated total scores.

#### Anxiety

Anxiety was evaluated by the School Anxiety Scale—Teacher Report (SAS-TR) from April 2017 to June 2018. It is a 16-item validated scale measured by teachers on a 4-point Likert scale that evaluates a student’s generalized and social anxiety over the past 3 months. Items include “This child worries about things” (generalized anxiety) and “This child seems very shy” (social anxiety), with the option to answer from “not at all,” “sometimes,” “often,” or “a great deal.” The final score for SAS-TR was calculated by taking the sum of each individual items’ score, with a possible score range of 0–48. It has been reported to have high internal consistency and has shown evidence of convergent and divergent validity (Lyneham et al., 2008). SAS-TR had excellent internal consistency at baseline in this sample, with a Cronbach’s alpha of 0.90.

The 8-item Spence Children’s Anxiety Scale (SCAS) was used to measure anxiety from October 2018 to the endpoint of data collection for this study. The brief SCAS has both parent and teacher versions and was designed to assess symptoms of DSM-IV anxiety disorders in children. Examples of items include “Worries about things” and “Worries what people think of him/her,” with possible answer choices being “never,” “sometimes,” “often,” and “always.” It has a final score range of 0–24, which is calculated by taking the sum of the individual items’ scores. The measure is known to have good internal consistency, agreement among reporters, and convergent and divergent validity (Reardon et al., 2018). In this sample, the 8-item SCAS had good and fair internal consistency at baseline for the parent- and teacher-reported versions respectively (Cronbach’s alpha: 0.81 and 0.72). The additional measure of parent ratings of anxiety that SCAS provided, when compared to SAS-TR, was one of the primary reasons for the switch in anxiety scales, as that would allow us to gage the child’s anxiety in their home environment. In addition, the 8-item SCAS was a much shorter questionnaire compared to SAS-TR, which we hoped would result in better completion rates. For both scales, higher scores indicated higher anxiety.

#### Depression

Depression was evaluated by the Short Mood and Feelings Questionnaire (SMFQ), which was evaluated by parents. This is a validated scale that assesses how a child has been feeling lately and is a screening tool for depression in children and young people aged 6 to 19. The scale was introduced from November 2018 onwards. It is a 13-item questionnaire, with items such as “S/he felt s/he was no good anymore” and “S/he hated him/herself,” that are answered on a 3-point Likert scale: “not true,” “sometimes,” or “true.” The final score range is 0–26 (calculated by taking the sum of the score for each individual item), with a higher score indicating increased levels of depression. The SMFQ has been shown to have good internal reliability (Angold et al., 1995) and content, convergent, and concurrent validity (Thabrew et al., 2018). The SMFQ had good internal consistency at baseline in this sample, with a Cronbach’s alpha of 0.85. As this measure was introduced as an outcome after the broader cohort study began, we do not have the baseline depression measures for the participants who enrolled before that timepoint, resulting in smaller sample sizes for this particular outcome.

#### Academic performance

Academic performance was measured by teacher ratings of academic progress. The academic progress scale consisted of questions in a number of areas, including reading, writing, math, and other skills. The three schools used slightly different academic progress scales, because they were based on the existing forms that the teachers already used in those schools for evaluation. However, the content was very similar to one another. The following domains were covered by all schools: reading, writing, language skills, math and science, and executive functioning, whereas communication reasoning and social emotional learning were covered in two schools. Each item pertained to the student’s competence on a particular academic topic (e.g., reading comprehension, word recognition, algebraic thinking, or achieving goals), with the option to indicate the student’s level for that academic criterion on a Likert scale (e.g., low skill level, skill development, increasing independence, consistent independence with some support, or consistent independence without support).

Each academic progress scale was scored in a similar manner, where the final score was the sum of each individual items’ score in the scale. This sum across multiple domains was done in order to get a holistic view of the child’s academic performance, since children with RD are known to face academic challenges in many domains and may face a decline in overall school performance (Koerte et al., 2016; Sanfilippo et al., 2020). School A’s academic progress scale had 29 questions, with a maximum possible final score of 116. All the questions were answered on a 5-point Likert scale. School B’s academic progress scale had 30 questions, with a maximum possible final score of 119. Twenty-seven questions were answered on a 5-point Likert scale, 2 questions were answered on a 4-point Likert scale and one question was answered on a 6-point Likert scale. School C’s academic progress scale had 21 questions, with a maximum possible final score of 63. All the questions were answered on a 4-point Likert scale. All academic progress scales showed excellent internal consistency at baseline, with Cronbach’s alpha of 0.94, 0.95, and 0.93 for Schools A, B, and C respectively.

#### Quality of life

Quality of life was measured by the validated Pediatric Quality of Life Inventory 4.0 (PedsQL) (Varni et al., 2001). It was reported by the parents. PedsQL evaluates the child’s health-related quality of life. It has five distinct versions designed for different age groups (2–4 years, 5–7 years, 8–12 years, 13–18 years, and 19 years and older). Each version has the same content but has slightly different wording in certain items to best suit the age range of the child. We used the first four versions of PedsQL in this study to suit the age range of our participants. It is a 23-item questionnaire comprised of four domains: physical functioning (8 items), emotional functioning (5 items), social functioning (5 items), and school functioning (5 items). Each question starts off with “In the past one month, how much of a problem has the child had with…”, with examples of items being “doing chores around the house,” “trouble sleeping,” “getting along with other children,” and “keeping up with schoolwork.” These are then answered on a 5-point Likert scale, with the options: “never,” “almost never,” “sometimes,” “often,” or “almost always.” The total scale score is calculated by taking the sum of each individual item’s score. PedsQL has a total scale score range of 0–100, with higher scores indicating better QOL. Studies have shown PedsQL to have acceptable psychometric properties, with good reliability and validity (Bastiaansen et al., 2004; Petersen et al., 2009; Varni et al., 2001). PedsQL had excellent internal consistency at baseline (Cronbach’s alpha: 0.91) in this sample.

### Statistical analysis

Since anxiety and academic performance were assessed using multiple scales at different time points and between the different schools over the course of the study, in order to combine the different measures and better interpret the results, we standardized all the survey total scores to obtain their *z* scores. In order to do this, we first calculated the mean and standard deviation (by combining all the longitudinal data) for each measure (i.e., Grit and Resilience—Parent, Grit and Resilience—Teacher, SAS-TR, SCAS—Teacher, SMFQ, academic progress scores for Schools A, B, and C, and PedsQL), including only those who had completed the relevant questionnaires. Then, for each datapoint for each measure, we calculated the *z* score by subtracting the mean from the raw score and dividing it by the standard deviation. As such, for each observation per participant, a normalized score for every measure was obtained at every timepoint. These *z* scores were thereafter used in all subsequent analyses.

Baseline characteristics were summarized using descriptive statistics. Pearson correlations were used to assess correlations among predictor and outcome variables at baseline. Prior to conducting the formal analysis for hypothesis testing, we first did an exploratory analysis to look at the change in the mean grit and resilience scores over time among all participants in order to determine whether these specialized schools were having an impact on their overall grit and resilience. Repeated measures analysis using mixed-effects models was used to determine whether there were significant changes in the mean grit and resilience across the timepoints, with “subject ID” and “school” being defined as random effects. In order to determine whether the number of available timepoints played a role in this trend, sensitivity analyses were done to look at the change in the mean grit and resilience scores over time in the following subgroups: (I) those with at least 4 timepoints of measurements, and (II) those with measurements at 3 timepoints or less.

We tested two key questions in this study. First, we investigated the association between grit and resilience (predictor variable) and all the outcomes (anxiety, depression, academic performance, and quality of life) cross-sectionally at baseline, with parent-reported and teacher-reported grit and resilience being analyzed separately. This was done using linear regression models, with each model being subsequently adjusted for the age and sex of the child. Therefore, a total of 8 univariate (unadjusted) regression models and 8 multivariate (adjusted) regression models were run. In order to determine if the nesting of students within the different school sites contributed to significant variability at baseline, we tested for its random effects by conducting the Bruesch-Pagan Lagrange multiplier test for all our models, which showed evidence for no significant difference across schools. As such, simple linear regression models were used for the cross-sectional analyses.

Second, we examined the association between the change in grit and resilience and the change in outcomes (anxiety, depression, academic performance, and quality of life) within an individual over time. Repeated measures analysis using linear mixed-effects models were used to assess this relationship. We used mixed-effects models to study longitudinal associations, instead of predicting change in outcomes with baseline grit and resilience, to use the full power of the dataset and take all available observations into account. Generating change scores for the alternate analysis (e.g., change in one year) involved losing a large number of observations (70% loss), which markedly decreased the power of the analysis. As such, we felt that this was not the most rigorous way to examine the true associations in the dataset.

We defined “subject ID” as the major source of random effects, while time was coded as an ordinal variable (as each timepoint of measurement, and therefore had a range of 1 to 10). We accounted for the nesting of students within the school sites by entering “school” as a random effect in the mixed-effects model. We subsequently controlled for the age and sex of the child in all models. Parent-reported and teacher-reported grit and resilience were analyzed separately. While the parent-rater for a given participant was constant throughout the study, participants who were enrolled in the study for longer periods of time had more than one teacher-rater, as they had a new teacher for each academic year. The number of teacher-raters ranged between one and four, depending on when the participant enrolled. In order to account for this change, we adjusted for the number of teacher-raters that a child had over the course of the study. A total of 8 unadjusted linear mixed-effects models and 8 adjusted linear mixed-effects models were run. We focused specifically on the within-subject association; to make sure that the measured change in variables was due to within-individual change rather than between-individual change, we decomposed the predictor variable (grit and resilience) to two components: the mean and deviation. We then entered both pieces into the model as predictors, where the mean form represented between-cluster association between grit and resilience and the outcome of interest and the deviation form represented the within-cluster association. All results of the mixed-effects models report the deviation form of grit and resilience to reflect the within-subject change. Mixed-effects models also have the advantage of controlling for differences in the number of timepoints of data available between the participants (due to staggered entry into the study). Since the predictor in all models was time-varying, we did not include any time variable into the models as the coefficients for the models can directly be interpreted as the change in outcome associated with the change in the predictor (Vittinghoff et al., 2012). There was no minimum number of available timepoints required for a participant to be included in this analysis.

The equations for the adjusted mixed-effect models are given below. The first one corresponds to all models with parent-reported grit and resilience as the primary predictor, whereas the second equation corresponds to models with teacher-reported grit and resilience as the primary predictor. In both cases, a full form of the equation and a shorter form are provided, where the parameters in the short form correspond to those in the full form in sequential order.

1 $${\displaystyle \begin{array}{c}{Y}_{\mathrm{Outcome}}={\beta}_0+{\beta}_{\mathrm{G}\&\mathrm{R}-\mathrm{Parent}\ \left(\mathrm{Mean}\ \mathrm{Form}\right)}\times {X}_{\mathrm{G}\&\mathrm{R}-\mathrm{Parent}\ \left(\mathrm{Mean}\ \mathrm{Form}\right)}\\ {}+{\beta}_{\mathrm{G}\&\mathrm{R}-\mathrm{Parent}\ \left(\mathrm{Deviation}\ \mathrm{Form}\right)}\times {X}_{\mathrm{G}\&\mathrm{R}-\mathrm{Parent}\ \left(\mathrm{Deviation}\ \mathrm{Form}\right)}\\ {}\begin{array}{c}+{\beta}_{\mathrm{Age}}\times {X}_{\mathrm{Age}}+{\beta}_{\mathrm{Sex}}\times {X}_{\mathrm{Sex}}+{\gamma}_{\mathrm{School}}\times {Z}_{\mathrm{School}}+{\gamma}_{\mathrm{Student}}\times {Z}_{\mathrm{Student}}+\epsilon \\ {}\begin{array}{c}\mathrm{which}\ \mathrm{can}\ \mathrm{alternatively}\ \mathrm{be}\ \mathrm{written}\ \mathrm{in}\ \mathrm{as}:\\ {}{Y}_{\mathrm{Outcome}}={\mu}_0+{\mu}_1+{\mu}_2+{\mu}_3+{\mu}_4+{\lambda}_1+{\lambda}_2+\epsilon \end{array}\end{array}\end{array}}$$

2 $${\displaystyle \begin{array}{c}\begin{array}{c}{Y}_{\mathrm{Outcome}}={\beta}_0+{\beta}_{\mathrm{G}\&\mathrm{R}-\mathrm{Teacher}\ \left(\mathrm{Mean}\ \mathrm{Form}\right)}\times {X}_{\mathrm{G}\&\mathrm{R}-\mathrm{Teacher}\ \left(\mathrm{Mean}\ \mathrm{Form}\right)}\\ {}+{\beta}_{\mathrm{G}\&\mathrm{R}-\mathrm{Teacher}\ \left(\mathrm{Deviation}\ \mathrm{Form}\right)}\times {X}_{\mathrm{G}\&\mathrm{R}-\mathrm{Teacher}\ \left(\mathrm{Deviation}\ \mathrm{Form}\right)}\\ {}+{\beta}_{\mathrm{Age}}\times {X}_{\mathrm{Age}}+{\beta}_{\mathrm{Sex}}\times {X}_{\mathrm{Sex}}+\kern0.5em {\beta}_{\mathrm{Number}\ \mathrm{of}\ \mathrm{Raters}}\times {X}_{\mathrm{Number}\ \mathrm{of}\ \mathrm{Raters}}+{\gamma}_{\mathrm{School}}\times {Z}_{\mathrm{School}}+{\gamma}_{\mathrm{Student}}\times {Z}_{\mathrm{Student}}+\epsilon \end{array}\\ {}\mathrm{which}\ \mathrm{can}\ \mathrm{alternatively}\ \mathrm{be}\ \mathrm{written}\ \mathrm{in}\ \mathrm{as}:\\ {}{Y}_{\mathrm{Outcome}}={\mu}_0+{\mu}_5+{\mu}_6+{\mu}_3+{\mu}_4+{\mu}_7+{\lambda}_1+{\lambda}_2+\epsilon \end{array}}$$

Models were assessed for linearity, normality, constant variance, and influential points. For models that showed apparent evidence of skewness (departure from normality), we used the nonparametric bootstrapping method of bias-corrected percentile confidence intervals (with 1000 bootstrap samples) to calculate the regression coefficients and confidence intervals. Sensitivity analysis was conducted on models that indicated possible influential points by removing these data points. In all cases, there were no qualitative differences after excluding them. Therefore, the original data was kept. P-values of less than 0.04 were considered statistically significant, after correcting for multiple comparisons using the Benjamini–Hochberg procedure, with a false discovery rate of 0.1 (McDonald, 2014). All analyses were performed using STATA and SAS.

## Results

### Baseline characteristics

Of the 163 participants in the cohort, males comprised 62% of the population (Table 1). The mean age (and SD) was 11 ± 3 years, with participants ranging between 6 and 16 years of age (Table 1). The number of participants that came from each school is also reported (Table 1). The mean ± SD and range of grit and resilience (parent and teacher) score, SAS-TR score, 8-item SCAS (parent and teacher) score, SMFQ score, academic performance scores, and PedsQL scores are shown in Table 2. The correlations matrix showed low to moderate correlations between the various predictors and outcome variables at baseline (Table 3).

**Table 1:** Sociodemographic characteristics of participants at baseline (N = 163)

	Full sample						School A						School B								School C				
	Mean	SD	Min	Max	N	%	Mean	SD	Min	Max	N	%	Mean	SD	Min	Max	N	%	Mean	SD		Min	Max	N	%
Demographics																									
School					163	100					104	63.8					30	18.4						29	17.8
Age (years)	11	3	6	16	162		10	2	6	14	103		11.5	1	7	14	30		12	3		9	16	29	
Sex																									
Male					101	62.4					61	59.2					22	73.3						18	62.1
Female					61	37.6					42	40.8					8	26.7						11	37.9

**Table 2:** Summary statistics of predictor and outcome measures at baseline

Measure	N	Mean	SD	Minimum	Maximum
Grit & resilience					
Grit & resilience—parent	139	40.9	10	12	62
Grit & resilience—teacher	152	50.6	15	10	79
Anxiety					
School Anxiety Scale—teacher report	52	10.2	8	0	34
8-item Spence Children’s Anxiety Scale—parent	99	6.2	3.5	0	18
8-item Spence Children’s Anxiety Scale—teacher	96	3.6	2.6	0	18
Depression					
Short Mood and Feelings Questionnaire (SMFQ)	101	3.4	3.8	0	19
Academic performance					
School A	97	71.9	20.4	27	125
School B	29	58.3	24.2	2	110
School C	25	34.2	10.7	14	54
Quality of life					
Pediatric quality of life	141	65.2	15.6	25	91

**Table 3:** Pearson correlation matrix for continuous variables at baseline

Baseline variable	1	2	3	4	5	6
1. Grit—parent	-					
2. Grit—teacher	0.51^***^	-				
3. Anxiety	− 0.27^**^	− 0.49^***^	−			
4. Depression	− 0.38^***^	− 0.37^***^	0.29^**^	−		
5. Academic performance	0.18^*^	0.52^***^	− 0.37^***^	− 0.21^*^	-	
6. Quality of life	0.32^***^	0.23^**^	− 0.19^*^	− 0.61^***^	0.18^*^	-

^*^p < 0.05; ^**^p < 0.01; ^***^p < 0.001

### Change in average grit and resilience over time

**Fig. 1:**
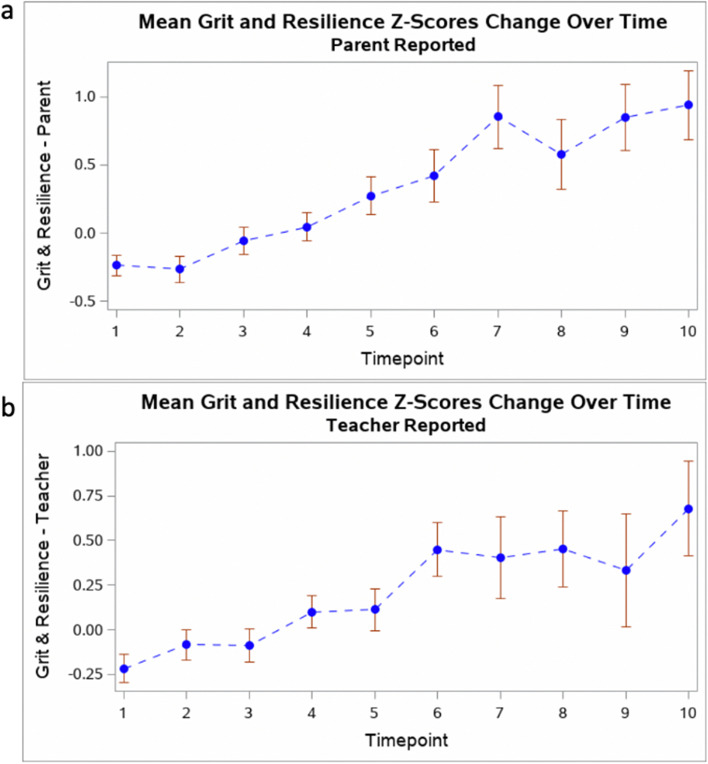
Mean normalized grit and resilience scores (with error bars), when measured by parents (**a**) and by teachers (**b**), among all participants at each timepoint, showing the overall change in mean grit and resilience over time

The average normalized grit and resilience scores among all participants steadily increased over time, regardless of whether it was evaluated by parents or by teachers (Fig. 1). The results from the mixed-effects model showed that mean grit and resilience significantly increased with time, both when measured by parents (*p* < 0.001) or by teachers (*p* < 0.001).

In our sensitivity analysis, we found that while the subgroup of participants who had at least four timepoints of data available (i.e., those who joined the study earlier) showed an increase in the mean grit and resilience over time (*p *< 0.001 for both parent- and teacher-reported), the subgroup of participants who had measurements at three points or less (i.e., those who joined the study more recently) did not show any such change (*p* = 0.5 for parent-reported and *p* = 0.2 for teacher-reported). These results indicate that the general increase in grit and resilience depends on how long one has been in the study, and that there is a certain time threshold to cross (approximately 1 year) before we see any significant increases in grit and resilience. Nevertheless, in general, there were statistically significant improvements in mean grit and resilience (as rated by both parents and teachers) over time.

### Baseline associations between grit and resilience and outcomes

**Fig. 2:**
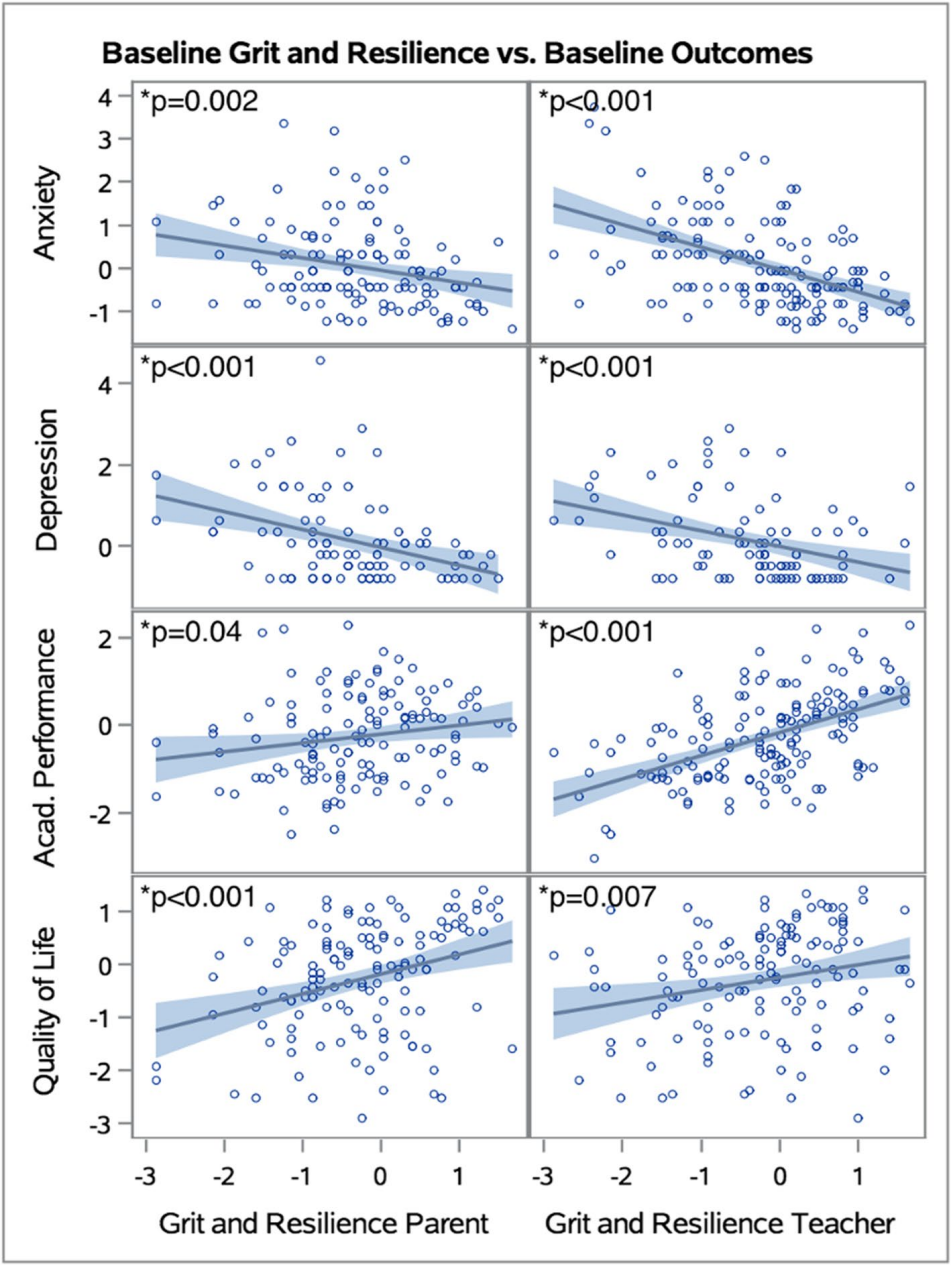
Univariate relationships between baseline grit and resilience and baseline mental health outcomes, academic performance, and quality of life. In each figure, as baseline normalized grit and resilience scores increase, the normalized outcome scores at baseline improve (i.e., lower anxiety, lower depression, higher academic performance, and higher quality of life)

Overall, there were statistically significant associations between grit and resilience and all the outcomes of interest at baseline (Table 4). In general, at baseline, those with higher grit and resilience scores were more likely to have better mental health (i.e., reduced anxiety and depression), higher levels of academic performance, and better quality of life (Fig. 2).

**Table 4:** Crude and adjusted associations between grit and resilience and all outcomes: anxiety, depression, academic performance, and quality of life at baseline

Measure		N	β	SE	95% CI		R^2^	*p*
					LL	UL		
Anxiety								
Grit & resilience—parent	Unadjusted	127	− 0.3	0.09	− 0.5	− 0.1	0.07	0.002*
	+ age, sex	127	− 0.3	0.09	− 0.5	− 0.1	0.09	0.001*
Grit & resilience—teacher	Unadjusted	147	− 0.5	0.08	− 0.7	− 0.4	0.24	< 0.001*
	+ age, sex	145	− 0.5	0.08	− 0.7	− 0.4	0.26	< 0.001*
Depression								
Grit & resilience—parent	Unadjusted	90	− 0.4	0.11	− 0.7	− 0.2	0.14	< 0.001*
	+ age, sex	90	− 0.5	0.11	− 0.7	− 0.2	0.20	< 0.001*
Grit & resilience—teacher	Unadjusted	92	− 0.4	0.10	− 0.6	− 0.2	0.13	< 0.001*
	+ age, sex	92	− 0.4	0.10	− 0.6	− 0.2	0.14	< 0.001*
Academic performance								
Grit & resilience—parent	Unadjusted	128	0.2	0.10	0.01	0.4	0.03	0.04
	+ age, sex	128	0.2	0.10	− 0.02	0.4	0.07	0.09
Grit & resilience—teacher	Unadjusted	151	0.5	0.07	0.4	0.7	0.27	< 0.001*
	+ age, sex	149	0.5	0.07	0.4	0.7	0.32	< 0.001*
Quality of life								
Grit & resilience—parent	Unadjusted	132	0.4	0.09	0.2	0.5	0.11	< 0.001*
	+ age, sex	132	0.4	0.10	0.2	0.6	0.12	< 0.001*
Grit & resilience—teacher	Unadjusted	132	0.2	0.09	0.06	0.4	0.05	0.009*
	+ age, sex	131	0.2	0.09	0.05	0.4	0.05	0.01*

*Note. LL* lower limit; *UL* upper limit; *p < 0.04

In unadjusted analyses, grit and resilience was significantly associated with anxiety, regardless of whether grit and resilience was measured by parents (*p* = 0.002) or by teachers (*p* < 0.001). These associations remained statistically significant even after controlling for age and sex (parent-reported: *p* = 0.001; teacher-reported: *p* < 0.001). Similarly, grit and resilience was significantly associated with depression in unadjusted analyses (parents-reported: *p *< 0.001; teacher-reported: *p* < 0.001). After adjusting for age and sex, these associations remained statistically significant (parent-reported: *p* < 0.001; teacher-reported: *p* < 0.001).

In unadjusted analyses, teacher-reported grit and resilience was significantly associated with academic performance (*p* < 0.001), but parent-reported grit and resilience was not (*p* = 0.04). After adjusting for age and sex, teacher-reported grit and resilience remained significantly associated with academic performance (*p* < 0.001). Similarly, grit and resilience was significantly associated with quality of life in unadjusted analyses (parents-reported: *p* < 0.001; teacher-reported: *p* = 0.009). These associations remained statistically significant even after controlling for age and sex, (parent-reported: *p* < 0.001; teacher-reported *p* = 0.01).

### Longitudinal associations between grit and resilience and outcomes

Overall, there was evidence of statistically significant associations between change in grit and resilience and change in all the outcomes of interest over time; however, the observed associations depended on how grit and resilience was measured (whether by parents or by teachers) for each outcome (Table 5). Nevertheless, we found that within an individual, improvements in grit and resilience were associated with improvements in mental health (i.e., reduction in anxiety and depression), in academic performance, and in quality of life over time.

**Table 5:** Crude and adjusted associations between grit and resilience and all outcomes (anxiety, depression, academic performance, and quality of life) over time within an individual

		Parameter	N	β	SE	95% CI		*p*
						LL	UL	
Anxiety	Intercept	μ_0_		− 0.02	0.07	− 0.1	0.1	0.7
	Grit & resilience**—**parent (unadjusted)	μ_2_	151	− 0.1	0.07	− 0.2	0.03	0.1
	Intercept	μ_0_		0.14	0.4	− 0.6	0.9	0.7
	Grit & resilience**—**parent (adjusted)^a^	μ_2_	150	− 0.1	0.06	− 0.2	0.02	0.1
	Intercept	μ_0_		− 0.03	0.05	− 0.5	− 0.3	0.6
	Grit & resilience**—**teacher (unadjusted)	μ_6_	160	− 0.4	0.05	− 0.5	− 0.3	< 0.001*
	Intercept	μ_0_		− 0.01	0.33	− 0.01	0.2	0.9
	Grit & resilience**—**teacher (adjusted)^b^	μ_6_	158	− 0.4	0.05	− 0.5	− 0.3	< 0.001*
Depression	Intercept	μ_0_		− 0.03	0.07	− 0.2	0.1	0.7
	Grit & resilience**—**parent (unadjusted)	μ_2_	134	− 0.3	0.08	− 0.5	− 0.1	0.002*
	Intercept	μ_0_		− 0.6	0.39	− 1.3	0.2	0.1
	Grit & resilience**—**parent (adjusted)^a^	μ_2_	133	− 0.3	0.08	− 0.5	− 0.1	0.001*
	Intercept	μ_0_		0.01	0.07	− 0.1	0.1	0.9
	Grit & resilience**—**teacher (unadjusted)	μ_6_	135	− 0.1	0.06	− 0.3	0.02	0.2
	Intercept	μ_0_		0.08	0.43	− 0.8	0.9	0.8
	Grit & resilience**—**teacher (adjusted)^b^	μ_6_	134	− 0.1	0.06	− 0.2	0.04	0.2
Academic performance	Intercept	μ_0_		0.05	0.07	− 0.09	0.2	0.5
	Grit & resilience**—**parent (unadjusted)	μ_2_	150	0.2	0.07	0.04	0.3	0.009*
	Intercept	μ_0_		− 2.2	0.39	− 2.9	− 1.4	< 0.001*
	Grit & resilience**—**parent (adjusted)^a^	μ_2_	149	0.1	0.06	− 0.03	0.2	0.15
	Intercept	μ_0_		0.1	0.1	− 0.08	0.3	0.2
	Grit & resilience**—**teacher (unadjusted)	μ_6_	162	0.5	0.05	0.4	0.6	< 0.001*
	Intercept	μ_0_		− 1.5	0.31	− 2.1	− 0.9	< 0.001*
	Grit & resilience**—**teacher (adjusted)^b^	μ_6_	160	0.5	0.04	0.4	0.6	< 0.001*
Quality of life	Intercept	μ_0_		0.05	0.07	− 0.09	0.2	0.5
	Grit & resilience**—**parent (unadjusted)	μ_2_	152	0.6	0.07	0.4	0.8	< 0.001*
	Intercept	μ_0_		− 0.7	0.32	− 1.3	− 0.2	0.04
	Grit & resilience**—**parent (adjusted)^a^	μ_2_	151	0.6	0.07	0.4	0.8	< 0.001*
	Intercept	μ_0_		− 0.02	0.06	− 0.1	0.1	0.8
	Grit & resilience**—**teacher (unadjusted)	μ_6_	153	0.01	0.07	− 0.1	0.1	0.9
	Intercept	μ_0_		− 1.5	0.41	− 2.3	− 0.7	< 0.001*
	Grit & resilience**—**teacher (adjusted)^b^	μ_6_	152	− 0.02	0.06	− 0.1	0.1	0.8

*Note.* ^a^Adjusted for age and sex; ^b^adjusted for age, sex, and number of raters

*LL* lower limit; *UL* upper limit

This table summarizes the results of 16 mixed-effects models, reporting two fixed-effect parameters (intercept and X_G&R (Deviation Form)_). For the full results of all models, please see Supplementary Tables 2–5

^*^p < 0.04

Change in teacher-reported grit and resilience was significantly associated with change in anxiety over time within an individual in both unadjusted analyses (regression coefficient or β =  − 0.4, *p* < 0.001), and after adjusting for age, sex, and number of teacher-raters (β =  − 0.4, *p* < 0.001). This means that within a subject, every SD increase in their grit and resilience was associated with a 0.4 SD decrease in their anxiety score over time. However, this association was not seen for parent-reported grit and resilience. Similarly, change in parent-reported grit and resilience was significantly associated with change in depression over time within an individual in both unadjusted analyses (β =  − 0.3, *p* < 0.001), and after adjusting for age and sex (β =  − 0.3, *p* < 0.001). However, this association was not seen for teacher-reported grit and resilience.

Change in teacher-reported grit and resilience was significantly associated with change in academic performance over time within an individual in both unadjusted analyses (β = 0.5, *p* < 0.001), and after adjusting for age, sex, and number of teacher-raters (β = 0.5, *p* < 0.001). However, this association was not seen for parent-reported grit and resilience. Similarly, parent-reported change in grit and resilience was significantly associated with change in quality of life over time within an individual, in both unadjusted analyses (β = 0.6, *p* < 0.001), and after adjusting for age and sex (β = 0.6, *p* < 0.001). However, this association was not seen for teacher-reported grit and resilience.

## Discussion

Grit and resilience are essential components to one’s success, with grit being related to persistence of effort and resilience being related to response to difficulties. Given their importance to attaining success, it seemed worthwhile to examine whether they were also predictive in children and adolescents with specific challenges, namely those with reading disorder (RD). In this 3-year longitudinal cohort study, we used a combined measure of grit and resilience and assessed its relationship with anxiety, depression, academic performance, and quality of life in children with RD, with the primary goal of determining if grit and resilience was associated with these other outcomes. In particular, we examined whether baseline grit and resilience could predict baseline outcomes and whether change in grit and resilience could predict change in outcomes over time.

Our primary results showed grit and resilience to be significantly associated with anxiety, depression, academic performance, and quality of life in children with RD, both cross-sectionally and longitudinally, even after adjusting for age and sex. If the direction of this association is accurate (i.e., grit and resilience leading to these outcomes), then these findings may suggest that interventions to improve grit and resilience are likely to lead to many positive benefits. Notably, we were able to demonstrate that on average grit and resilience improved over time, suggesting that the specialized education environment in these dyslexia schools is fostering a positive change. This finding is of particular interest because grit and resilience have been argued to have trait-like characteristics (Usher et al., 2018), with mixed results regarding their response to educational interventions that aim to improve them (Stoffel & Cain, 2018). Our results suggest that grit and resilience may be amenable to improvement (at least in the RD population) as a result of the efforts of these specialized schools after a certain threshold. However, it should be noted that while the magnitude of change was large (effect size > 0.8) and suggested clinical significance (Kraemer et al., 2003), since this is a novel scale, further research is required to determine whether this magnitude of difference is practically meaningful.

We believe that our results provide compelling new evidence that improved grit and resilience is positively associated with improved mental health, academic performance, and quality of life in young population with RD. Not only were the associations between grit and resilience and the outcomes of interest statistically significant, the magnitude of their effect sizes is also of high importance. The absolute value for the effect sizes ranged between 0.3 and 0.6 in the longitudinal analysis (0.4 for anxiety, 0.3 for depression, 0.5 for academic performance, and 0.6 for quality of life), which indicates a moderate effect of grit and resilience on all the outcomes.

While longitudinal analysis showed improvements in grit and resilience to be associated with improvements in outcomes within an individual, it should be noted that the observed associations depended on the evaluator of grit and resilience, that is whether it was measured by parents or by teachers. Notably, increased grit and resilience was significantly associated with decreased depression and increased quality of life when grit and resilience was measured by parents (but not by teachers). Conversely, increased grit and resilience was significantly associated with decreased anxiety and increased academic performance when grit and resilience was measured by teachers (but not by parents). Interestingly enough, depression and quality of life were evaluated by parents, whereas anxiety and academic performance were evaluated by teachers, respectively. This indicates that these significant associations only appeared when both the predictor and outcome were being measured by the same evaluator. While the issue of measurement bias may come to mind, there is no reason to believe that either the parents or the teachers would have systematic bias (e.g., reporting a lower anxiety/depression score when they observe a greater level of grit and resilience).

Rather, these results suggest the possibility that grit and resilience may have different qualities depending on the context and environment, i.e., grit and resilience in the home environment (which is measured by parents) may be inherently different from grit and resilience in the classroom environment (which is measured by the teachers). This could explain why grit and resilience in the home environment was only associated with other outcomes observed and evaluated in the same environment by the same evaluators (depression and quality of life) but was not associated with outcomes seen in the classroom environment (anxiety and academic performance). Similarly, this may explain why grit and resilience in the classroom environment was only associated with other outcomes measured in the classroom environment (anxiety and academic performance) but was not associated with outcomes seen in the classroom environment (depression and quality of life). Further research is required to delineate how grit and resilience may be manifested in different environments and contexts and whether those variances are significant enough to classify them as different constructs.

There has been much research on how grit and/or resilience is related to mental health, academic achievement, and quality of life in other populations that do not have RD, which indicate the positive impact of both grit and resilience on all these outcomes in the general population. However, to our knowledge, grit and resilience had not previously been examined in the RD population, especially in the context of these outcomes. Our findings are consistent with previous studies by showing higher levels of grit and resilience to be associated with reductions in anxiety and depression and improvements in quality of life. In the case of academic performance, our results are consistent with studies that show a moderate positive association between grit and academic performance. One reason why we may have seen a stronger relationship, in contrast to studies that showed weak associations or indeed did not find any such associations as all, may be because of the nature of the performance domain, which has been proposed to play a moderating role in the relationship. In essence, high levels of grit may have the most impact on academic performance, if the task is difficult and well-defined, whereas grit may not be as useful for easy or novel and ill-defined tasks (Credé et al., 2017). Perhaps because the RD population faces additional academic challenges, they benefit more from grit’s impact on academic performance. An alternative reason why we found a stronger association may be that our combined measure of grit and resilience may have focused more on perseverance of effort, which as previously mentioned has been shown to be a more powerful predictor of academic performance compared to consistency of interest.

Furthermore, the findings from this study also build upon prior knowledge by showing evidence for all these associations in a new population, thereby demonstrating the importance of grit and resilience in the RD population. Additionally, this result is interesting considering that many studies have shown the importance of a related construct, task-focused behavior, on literacy and reading achievement. Indeed, increased task-focused behavior has been shown to be a significant predictor of later reading fluency, letter knowledge, and phonological sensitivity in one study (Stephenson et al., 2008), and of later reading comprehension and spelling skills in another (Hirvonen et al., 2009). Task-focused behavior has also been shown to be a protective factor against developing RD among at-risk children (Eklund et al., 2013). In future studies, it would be interesting to see if grit and resilience play a similar protective role.

The longitudinal nature of this study is also of significance, as the majority of prior studies used cross-sectional designs and investigated outcomes at one particular point in time rather than examining the long-term effects of grit and resilience. Indeed, a systematic review about the role of grit in education commented that “no study explored whether goals were meaningful to participants instead of perceived as merely an endpoint in the educational process” (Christopoulou et al., 2018). Showing the positive association of improved grit and resilience with improvements in mental health and quality of life (as well as academic performance) in these participants contributes to close this gap in knowledge.

While there may be a concern for regression to the mean due to the current sample of children with RD being from specialized schools, comparisons of the mean anxiety (SAS-TR and 8-item SCAS) and depression (SMFQ) measures at both baseline and final timepoints in the current sample found them to be very similar to a normative typically developing population. In the case of SAS-TR, the baseline and final timepoint mean ± SD were 10.2 ± 8 and 10.5 ± 7, respectively, which is comparable to that in the typically developing population, 10.8 ± 8.4 (Lyneham et al., 2008). Similarly, parent-reported 8-item SCAS (baseline: 6.2 ± 3.5; final: 5.5 ± 3.4) in this sample was comparable to the typically developing population, 5.7 ± 3.7, and teacher-reported 8-item SCAS (baseline: 3.4 ± 3.8; final: 3.6 ± 3.9) in this sample was also comparable to the normative population, 3.4 ± 2.9 (Reardon et al., 2018). Depression scores (SMFQ) in this sample (baseline: 3.4 ± 3.8; final: 3.6 ± 3.9) were slightly higher than that in the typically developing population (3.2 ± 3.7) but arguably comparable nonetheless (Lerthattasilp et al., 2020). However, the average level of quality of life (PedsQL) at baseline (65.2 ± 15.6) and end of the study (72 ± 13.8) were both lower than that in the normative population (80.2 ± 15.9) (Varni et al., 2001), which raises concern of whether change over time was due to regression to the mean for quality of life specifically. In addition, since we do not have such comparable measures in the typically developing population for the measures with newly created scales (grit and resilience and academic performance), we cannot comment on how regression to the mean may have affected them.

This study has several limitations that should be noted. Firstly, although grit and resilience are strongly related constructs, so far, they have been studied as individual and separate concepts. As such, the combination of the two constructs together and its subsequent measurement by the Grit and Resilience Scale makes our variable unique and may hinder comparisons with other studies, especially considering that it did not factor as one construct. Nevertheless, grit and resilience are highly similar constructs and many items in the questionnaire were adapted from validated scales for the two separate measures (Connor & Davidson, 2003; Duckworth & Quinn, 2009), after consultation with teachers who specifically picked items that they believed to be relevant to children with RD. We believe that this helped improve their response and investment in the study. Another limitation is that two different anxiety measures (SAS-TR and 8-item SCAS) were used during the study, requiring the scores to be normalized to their *z* scores in order to allow comparison between the two. However, both scales had similar content and are both well-validated measures of the same clinical construct. Similarly, an additional limitation was the use of slightly different academic progress scales between the three schools, which may have had some impact on the summary *z* scores. Another limitation is that our current dataset does not allow for determination of directionality of the associations. Therefore, while this study focused on grit and resilience as the predictor, it is possible that the directionality of the relationship is reversed (whereby changes in the outcomes led to changes in grit and resilience), or that it goes in both directions. Finally, because this is an observational study, the associations observed between grit and resilience and all outcomes of interest may be susceptible to unmeasured confounding, thus limiting our ability to assess causality. Variables, such as classroom environment, self-esteem, interpersonal relationships, and severity of RD, may be linked to grit and resilience and all outcomes. However, considering that there is no consensus on whether these are confounders to begin with, or whether they could be mediators instead, we thought it prudent to not control for them. Nonetheless, we adjusted for age and sex using multivariate models.

The results from this study provide strong evidence that grit and resilience is significantly related to mental health, academic success, and quality of life in children with RD. The fact that these associations were seen both in cross-sectional analyses (at baseline) and in longitudinal analyses further strengthens this evidence. In addition, the increase in grit and resilience over time in all three schools demonstrates that a specialized school setting may be effective for improving grit and resilience, which may have set the stage for further improvements in other outcomes, such as mental health, academic performance, and quality of life. In future studies, intervention programs specifically targeted to increase grit and resilience may prove to be beneficial to the RD population. In order to do so, we could investigate the specific interventions used in these specialized schools and identify which ones were most predictive of changes in grit and resilience over time. This knowledge would then allow for an evidence-based program designed to specifically enhance grit and resilience. This program could then be further examined to see if implementation in a new school environment would lead to improvements in the secondary outcomes of mental health, academic performance, and quality of life.

### Supplementary Information

Below is the link to the electronic supplementary material.

Supplementary file1 (DOCX 35 KB)

## Data Availability

Not applicable.

## Appendix 1. Grit and Resilience Scale—parent

Please indicate how often [Child] does the following and answer with the options:

- Almost never
- Occasionally
- Sometimes
- Often
- Always almost

1. Articulates strengths and challenges.
2. Articulates to others how he/she/they learns best.
3. Transitions appropriately between tasks^1^.
4. Takes action to complete challenging tasks^1, 2^
5. Initiates and completes tasks^1^.
6. Uses strategies and tools to regain or maintain focus^1^.
7. Utilizes strategies to cope with challenging situations^2^.
8. Expresses positive self-perception^1, 2^
9. Accepts others' strengths, skills, and opinions.
10. Maintains positive, healthy peer relationships.
11. Adapts when changes occur^2^.
12. Bounces back after illness, injury, or other hardship^2^.
13. Stays focused and thinks clearly under pressure^2^.
14. Becomes easily discouraged by failure2^2^.
15. Advocates for personal needs.
16. Is willing to receive constructive feedback.
17. Accepts responsibility for he/she/they did.

^1^Adapted from and/or thematically similar to the 12-item Grit Scale.

^2^Adapted from and/or thematically similar to the 10-item CD-RISC.

## Appendix 2. Grit and Resilience Scale—teacher

Please indicate how often [Child] does the following and answer with the options:

- Almost never
- Occasionally
- Sometimes
- Often
- Always almost

1. Articulates strengths and challenges.
2. Articulates to others how he/she/they learn best.
3. Transitions appropriately between tasks^1^.
4. Takes action to complete challenging tasks^1, 2^
5. Initiates and completes tasks^1^.
6. Uses strategies and tools to regain or maintain focus^1^.
7. Participates appropriately during class discussions.
8. Utilizes strategies to cope with challenging situations^2^.
9. Expresses positive self-perception^1, 2^
10. Contributes positively to classroom environment.
11. Accepts others' strengths, skills, and opinions.
12. Works well in groups.
13. Maintains positive, healthy peer relationships.
14. Adapts when changes occur^2^.
15. Bounces back after illness, injury, or other hardships^2^.
16. Stays focused and thinks clearly under pressure^2^.
17. Becomes easily discouraged by failure^2^.
18. Advocates for educational needs.
19. Advocates for personal needs.
20. Is willing to receive constructive feedback.
21. Accepts responsibility for he/she/they did.

^1^Adapted from and/or thematically similar to the 12-item Grit Scale.

^2^Adapted from and/or thematically similar to the 10-item CD-RISC.

## References

[CR1] Angold A, Costello E, Messer S, Pickles A, Winder F, Silver D (1995). The development of a questionnaire for use in epidemiological studies of depression in children and adolescents. International Journal of Methods in Psychiatric Research.

[CR2] Askeland KG, Hysing M, Aarø LE, Tell GS, Sivertsen B (2015). Mental health problems and resilience in international adoptees: Results from a population-based study of Norwegian adolescents aged 16–19 years. Journal of Adolescence.

[CR3] Bastiaansen, D., Koot, H. M., Bongers, I. L., Varni, J. W., & Verhulst, F. C. (2004). Measuring quality of life in children referred for psychiatric problems: psychometric properties of the PedsQL 4.0 generic core scales. *Quality of Life Research, 13*(2), 489–495. 10.1023/B:QURE.0000018483.01526.ab 10.1023/B:QURE.0000018483.01526.ab 15085921

[CR4] Calvete E, Las Hayas C, Gómez Del Barrio A (2018). Longitudinal associations between resilience and quality of life in eating disorders. Psychiatry Research.

[CR5] Campbell-Sills L, Stein MB (2007). Psychometric analysis and refinement of the Connor-davidson Resilience Scale (CD-RISC): Validation of a 10-item measure of resilience. Journal of Traumatic Stress.

[CR6] Cederlof M, Maughan B, Larsson H, D'Onofrio BM, Plomin R (2017). Reading problems and major mental disorders - co-occurrences and familial overlaps in a Swedish nationwide cohort. Journal of Psychiatric Research.

[CR7] Christopoulou M, Lakioti A, Pezirkianidis C, Karakasidou E, Stalikas A (2018). The role of grit in education: A systematic review. Psychology.

[CR8] Connor KM, Davidson JR (2003). Development of a new resilience scale: The Connor-Davidson Resilience Scale (CD-RISC). Depression and Anxiety.

[CR9] Conway AM, McDonough SC (2006). Emotional resilience in early childhood: Developmental antecedents and relations to behavior problems. Annals of the New York Academy of Sciences.

[CR10] Credé M, Tynan MC, Harms PD (2017). Much ado about grit: A meta-analytic synthesis of the grit literature. Journal of Personality and Social Psychology.

[CR11] Datu JA, Valdez J, Eala M (2018). Grit is associated with lower depression via meaning in life among Filipino high school students. Youth & Society.

[CR12] Dray, J., Bowman, J., Campbell, E., Freund, M., Wolfenden, L., Hodder, R. K., . . . Wiggers, J. (2017). Systematic review of universal resilience-focused interventions targeting child and adolescent mental health in the school setting. *Journal of the American Academy of Child and Adolescent Psychiatry, 56*(10), 813–824. 10.1016/j.jaac.2017.07.780 10.1016/j.jaac.2017.07.780 28942803

[CR13] Duckworth AL, Peterson C, Matthews MD, Kelly DR (2007). Grit: Perseverance and passion for long-term goals. Journal of Personality and Social Psychology.

[CR14] Duckworth AL, Quinn PD (2009). Development and validation of the short grit scale (grit-s). Journal of Personality Assessment.

[CR15] Eklund KM, Torppa M, Lyytinen H (2013). Predicting reading disability: Early cognitive risk and protective factors. Dyslexia.

[CR16] Firth N, Frydenberg E, Steeg C, Bond L (2013). Coping successfully with dyslexia: An initial study of an inclusive school-based resilience programme. Dyslexia.

[CR17] Haft SL, Myers CA, Hoeft F (2016). Socio-emotional and cognitive resilience in children with reading disabilities. Current Opinion in Behavioral Sciences.

[CR18] Hirvonen R, Georgiou G, Lerkkanen M-K, Aunola K, Nurmi J-E (2009). Task-focused behaviour and literacy development: A reciprocal relationship. Journal of Research in Reading.

[CR19] Hjemdal O, Aune T, Reinfjell T, Stiles TC, Friborg O (2007). Resilience as a predictor of depressive symptoms: A correlational study with young adolescents. Clinical Child Psychology and Psychiatry.

[CR20] Hu T, Zhang D, Wang J-L (2015). A meta-analysis of the trait resilience and mental health. Personality and Individual Differences.

[CR21] Kim M, Kim K, Kim JS (2019). Impact of resilience on the health-related quality of life of adolescents with a chronic health problem: A structural equation approach: Resilience and health-related quality of life of adolescents. Journal of Advanced Nursing.

[CR22] King L, Jolicoeur-Martineau A, Laplante DP, Szekely E, Levitan R, Wazana A (2021). Measuring resilience in children: A review of recent literature and recommendations for future research. Current Opinion in Psychiatry.

[CR23] Koerte, I. K., Willems, A., Muehlmann, M., Moll, K., Cornell, S., Pixner, S., . . . Schulte-Korne, G. (2016). Mathematical abilities in dyslexic children: a diffusion tensor imaging study. *Brain Imaging and Behavior, 10*(3), 781–791. 10.1007/s11682-015-9436-y 10.1007/s11682-015-9436-y 26286825

[CR24] Kraemer HC, Morgan GA, Leech NL, Gliner JA, Vaske JJ, Harmon RJ (2003). Measures of clinical significance. Journal of the American Academy of Child and Adolescent Psychiatry.

[CR25] Lavin Venegas, C., N. Nkangu, M., Duffy, M., Fergusson, D., & Spilg, E. (2019). Interventions to improve resilience in physicians who have completed training: A systematic review. *PLoS One, 14*. 10.1371/journal.pone.0210512 10.1371/journal.pone.0210512 PMC6336384 30653550

[CR26] Lechner CM, Danner D, Rammstedt B (2019). Grit (effortful persistence) can be measured with a short scale, shows little variation across socio-demographic subgroups, and is associated with career success and career engagement. Plos One.

[CR27] Lerthattasilp T, Tapanadechopone P, Butrdeewong P (2020). Validity and reliability of the Thai version of the Short Mood and Feelings Questionnaire. East Asian Arch Psychiatry.

[CR28] Lyneham HJ, Street AK, Abbott MJ, Rapee RM (2008). Psychometric properties of the School Anxiety Scale-Teacher Report (SAS-TR). Journal of Anxiety Disorders.

[CR29] McBurnett K, Villodas M, Burns GL, Hinshaw SP, Beaulieu A, Pfiffner LJ (2014). Structure and validity of sluggish cognitive tempo using an expanded item pool in children with attention-deficit/hyperactivity disorder. Journal of Abnormal Child Psychology.

[CR30] McDonald JH (2014). Handbook of biological statistics.

[CR31] Muenks K, Wigfield A, Yang JS, O'Neal CR (2017). How true is grit? Assessing its relations to high school and college students' personality characteristics, self-regulation, engagement, and achievement. Journal of Education & Psychology.

[CR32] Muenks K, Yang J, Wigfield A (2017). Associations between grit, motivation, and achievement in high school students. Motivation Science.

[CR33] Musumari PM, Tangmunkongvorakul A, Srithanaviboonchai K, Techasrivichien T, Suguimoto SP, Ono-Kihara M, Kihara M (2018). Grit is associated with lower level of depression and anxiety among university students in Chiang Mai, Thailand: A cross-sectional study. Plos One.

[CR34] Petersen, S., Hägglöf, B., Stenlund, H., & Bergström, E. (2009). Psychometric properties of the Swedish PedsQL, Pediatric Quality of Life Inventory 4.0 generic core scales. *Acta Paediatrica, 98*(9), 1504–1512. 10.1111/j.1651-2227.2009.01360.x 10.1111/j.1651-2227.2009.01360.x 19570060

[CR35] Peterson RL, Pennington BF (2015). Developmental dyslexia. Annual Review of Clinical Psychology.

[CR36] Popa-Velea O, Diaconescu L, Jidveian Popescu M, Truţescu C (2017). Resilience and active coping style: Effects on the self-reported quality of life in cancer patients. International Journal of Psychiatry in Medicine.

[CR37] Reardon T, Spence SH, Hesse J, Shakir A, Creswell C (2018). Identifying children with anxiety disorders using brief versions of the Spence Children's Anxiety Scale for children, parents, and teachers. Psychological Assessment.

[CR38] Ríos-Risquez MI, García-Izquierdo M, Sabuco-Tebar E, Carrillo-Garcia C, Solano-Ruiz C (2018). Connections between academic burnout, resilience, and psychological well-being in nursing students: A longitudinal study. Journal of Advanced Nursing.

[CR39] Sanfilippo, J., Ness, M., Petscher, Y., Rappaport, L., Zuckerman, B., & Gaab, N. (2020). Reintroducing dyslexia: early identification and implications for pediatric practice. *Pediatrics*. 10.1542/peds.2019-3046 10.1542/peds.2019-3046 PMC7329249 32576595

[CR40] Sevincok D, Ozbay HC, Ozbek MM, Tunagur MT, Aksu H (2020). ADHD symptoms in relation to internalizing and externalizing symptoms in children: The mediating role of sluggish cognitive tempo. Nordic Journal of Psychiatry.

[CR41] Sharkey CM, Bakula DM, Gamwell KL, Mullins AJ, Chaney JM, Mullins LL (2017). The role of grit in college student health care management skills and health-related quality of life. Journal of Pediatric Psychology.

[CR42] Stephenson K, Parrila R, Georgiou G, Kirby J (2008). Effects of home literacy, parents' beliefs, and children's task-focused behavior on emergent literacy and word reading skills. Scientific Studies of Reading.

[CR43] Stoffel JM, Cain J (2018). Review of grit and resilience literature within health professions education. American Journal of Pharmaceutical Education.

[CR44] Straus E, Dev SI, Moore RC (2020). The measurement of resilience and grit: Room for improvement. Psychiatry Research.

[CR45] Suzuki, K., Kobayashi, T., Moriyama, K., Kaga, M., Hiratani, M., Watanabe, K., . . . Inagaki, M. (2015). Development and evaluation of a Parenting Resilience Elements Questionnaire (PREQ) measuring resiliency in rearing children with developmental disorders. *Plos One, 10*(12), e0143946. 10.1371/journal.pone.0143946 10.1371/journal.pone.0143946 PMC4669138 26633810

[CR46] Tang X, Wang MT, Guo J, Salmela-Aro K (2019). Building grit: The longitudinal pathways between mindset, commitment, grit, and academic outcomes. Journal of Youth and Adolescence.

[CR47] Tavakol M, Dennick R (2011). Making sense of Cronbach's alpha. International Journal of Medical Education.

[CR48] Thabrew H, Stasiak K, Bavin L-M, Frampton C, Merry S (2018). Validation of the Mood and Feelings Questionnaire (MFQ) and Short Mood and Feelings Questionnaire (SMFQ) in New Zealand help-seeking adolescents. International Journal of Methods in Psychiatric Research.

[CR49] Usher, E., Li, C., Butz, A., & Rojas, J. (2018). Perseverant grit and self-efficacy: are both essential for children's academic success? *Journal of Educational Psychology*. 10.1037/edu0000324

[CR50] Varni, J. W., Seid, M., & Kurtin, P. S. (2001). PedsQL 4.0: reliability and validity of the Pediatric Quality of Life Inventory version 4.0 generic core scales in healthy and patient populations. *Medical Care, 39*(8), 800–812. 10.1097/00005650-200108000-00006 10.1097/00005650-200108000-00006 11468499

[CR51] Vittinghoff, E., Glidden, D. V., Shiboski, S. C., & McCulloch, C. E. (2012). *Regression methods in biostatistics: linear, logistic, survival, and repeated measures models* (2nd ed.). Springer.

[CR52] Worku BN, Urgessa D, Abeshu G (2019). Psychosocial conditions and resilience status of street children in Jimma Town. Ethiopian Journal of Health Sciences.

[CR53] Young C, Craig JC, Clapham K, Banks S, Williamson A (2019). The prevalence and protective factors for resilience in adolescent Aboriginal Australians living in urban areas: A cross-sectional study. Australian and New Zealand Journal of Public Health.

[CR54] Zhang H, Zhao Q, Cao P, Ren G (2017). Resilience and quality of life: Exploring the mediator role of social support in patients with breast cancer. Med Sci Monit.

[CR55] Zhang, M. X., Mou, N. L., Tong, K. K., & Wu, A. M. S. (2018). Investigation of the effects of purpose in life, grit, gratitude, and school belonging on mental distress among Chinese emerging adults. *International Journal of Environmental Research and Public Health*. 10.3390/ijerph15102147 10.3390/ijerph15102147 PMC6210347 30274292

