# Supplementary material for: The role of grit and resilience in children with reading disorder: a longitudinal cohort study
Source: Ann Dyslexia. 2021 Jul 29;72(1):1–27. doi: 10.1007/s11881-021-00238-w (PMC8898035; doi:10.1007/s11881-021-00238-w)
Supplement: Supplementary file 1 — Supplementary file1 (DOCX 35 KB) [file 11881_2021_238_MOESM1_ESM.docx]

**Supplementary Table 1**

*Data availability for participants (N=163)*

| Number of Available Timepoints of Data | N |  | % |  |
| --- | --- | --- | --- | --- |
| 1 | 3 | 1.8 | | |
| 2 | 31 | 19 | | |
| 3 | 25 | 15.3 | | |
| 4 | 27 | 16.6 | | |
| 5 | 45 | 27.6 | | |
| 6 | 16 | 9.8 | | |
| 8 | 5 | 3.1 | | |
| 10 | 11 | 6.8 | | |

**Supplementary Table 2**

*Model parameters for mixed-effect models predicting Anxiety*

|  | Para  meter | Model 1 | | | Model 2 | | | Model 3 | | | Model 4 | | |
| --- | --- | --- | --- | --- | --- | --- | --- | --- | --- | --- | --- | --- | --- |
| Effect |  | Estimate | SE | 95% CI | Estimate | SE | 95% CI | Estimate | SE | 95% CI | Estimate | SE | 95% CI |
|  | | Fixed Effects | | |  | | | | | | | | |
| Intercept | μ_0_ | -0.02 | 0.07 | -0.2 – 0.1 | 0.14 | 0.4 | -0.6 – 0.9 | -0.03 | 0.05 | -0.1 – 0.08 | -0.01 | 0.3 | -0.6 – 0.6 |
| G&R-Parent (Mean) | μ_1_ | -0.19^**^ | 0.07 | -0.3 – -0.05 | -0.19^**^ | 0.07 | -0.3 – -0.05 |  |  |  |  |  |  |
| G&R-Parent (Deviation) | μ_2_ | -0.1 | 0.07 | -0.2 – 0.03 | -0.11 | 0.07 | -0.2 – 0.02 |  |  |  |  |  |  |
| G&R-Teacher (Mean) | μ_5_ |  |  |  |  |  |  | -0.4^***^ | 0.06 | -0.5 – -0.3 | -0.4^***^ | 0.06 | -0.5 – -0.3 |
| G&R-Teacher (Deviation) | μ_6_ |  |  |  |  |  |  | -0.4^***^ | 0.05 | -0.5 – -0.3 | -0.4^***^ | 0.05 | -0.5 – -0.3 |
| Age | μ_3_ |  |  |  | 0.01 | 0.03 | -0.05 – 0.1 |  |  |  | 0.02 | 0.02 | -0.03 – 0.06 |
| Male | μ_4_ |  |  |  | -0.17 | 0.14 | -0.4 – 0.09 |  |  |  | -0.3^*^ | 0.11 | -0.5 – -0.06 |
| Number of raters | μ_7_ |  |  |  |  |  |  |  |  |  | 0.1 | 0.06 | -0.01 – 0.2 |
|  | | Random Effects | | |  | | | | | | | | |
| Variance Components |  |  |  |  |  |  |  |  |  |  |  |  |  |
| School | λ_1_ | 0 |  |  | 0 |  |  | 0 |  |  | 0 |  |  |
| Student | λ_2_ | 0.44 | 0.07 | 0.3 – 0.6 | 0.45 | 0.08 | 0.3 – 0.6 | 0.3 | 0.05 | 0.2 – 0.4 | 0.29 | 0.05 | 0.2 – 0.4 |

*Note.* G&R = Grit and Resilience.

^*^*p*<0.05; ^**^*p*<0.01; ^***^*p*<0.001

**Supplementary Table 3**

*Model parameters for mixed-effect models predicting Depression*

|  | Para  meter | Model 1 | | | Model 2 | | | Model 3 | | | Model 4 | | |
| --- | --- | --- | --- | --- | --- | --- | --- | --- | --- | --- | --- | --- | --- |
| Effect |  | Estimate | SE | 95% CI | Estimate | SE | 95% CI | Estimate | SE | 95% CI | Estimate | SE | 95% CI |
|  | | Fixed Effects | | |  | | | | | | | | |
| Intercept | μ_0_ | -0.03 | 0.07 | -0.2 – 0.1 | -0.6 | 0.4 | -1.3 – 0.2 | 0.01 | 0.07 | -0.1 – 0.15 | 0.08 | 0.4 | -0.8 – 0.9 |
| G&R-Parent (Mean) | μ_1_ | -0.6^***^ | 0.07 | -0.7 – -0.4 | -0.6^***^ | 0.07 | -0.8 – -0.5 |  |  |  |  |  |  |
| G&R-Parent (Deviation) | μ_2_ | -0.3^***^ | 0.08 | -0.5 – -0.1 | -0.3^***^ | 0.08 | -0.5 – 0.2 |  |  |  |  |  |  |
| G&R-Teacher (Mean) | μ_5_ |  |  |  |  |  |  | -0.4^***^ | 0.08 | -0.6 – -0.3 | -0.4^***^ | 0.08 | -0.6 – -0.3 |
| G&R-Teacher (Deviation) | μ_6_ |  |  |  |  |  |  | -0.1 | 0.06 | -0.2 – 0.03 | -0.1 | 0.06 | -0.2 – 0.03 |
| Age | μ_3_ |  |  |  | 0.07 | 0.03 | 0.01 – 0.1 |  |  |  | 0.003 | 0.03 | -0.06 – 0.07 |
| Male | μ_4_ |  |  |  | -0.13 | 0.14 | -0.4 – 0.1 |  |  |  | 0.09 | 0.15 | -0.2 – 0.4 |
| Number of raters | μ_7_ |  |  |  |  |  |  |  |  |  | -0.12 | 0.08 | -0.3 – 0.03 |
|  | | Random Effects | | |  | | | | | | | | |
| Variance Components |  |  |  |  |  |  |  |  |  |  |  |  |  |
| School | λ_1_ | 0 |  |  | 0 |  |  | 0.001 | 0.02 |  | 0.003 | 0.03 |  |
| Student | λ_2_ | 0.44 | 0.08 | 0.3 – 0.6 | 0.44 | 0.08 | 0.3 – 0.6 | 0.45 | 0.08 | 0.3 – 0.6 | 0.45 | 0.08 | 0.3 – 0.7 |

*Note.* G&R = Grit and Resilience.

^*^*p*<0.05; ^**^*p*<0.01; ^***^*p*<0.001

**Supplementary Table 4**

*Model parameters for mixed-effect models predicting Academic Performance*

|  | Para  meter | Model 1 | | | Model 2 | | | Model 3 | | | Model 4 | | |
| --- | --- | --- | --- | --- | --- | --- | --- | --- | --- | --- | --- | --- | --- |
| Effect |  | Estimate | SE | 95% CI | Estimate | SE | 95% CI | Estimate | SE | 95% CI | Estimate | SE | 95% CI |
|  | | Fixed Effects | | |  | | | | | | | | |
| Intercept | μ_0_ | 0.05 | 0.07 | -0.1 – 0.2 | -2.2^***^ | 0.4 | -2.9 – -1.4 | 0.12 | 0.1 | -0.1 – 0.3 | -1.5^***^ | 0.3 | -2.1 – -0.9 |
| G&R-Parent (Mean) | μ_1_ | 0.24^**^ | 0.07 | 0.1 – 0.4 | 0.14 | 0.07 | -0.004 – 0.3 |  |  |  |  |  |  |
| G&R-Parent (Deviation) | μ_2_ | 0.18^**^ | 0.08 | 0.04 – 0.3 | 0.09 | 0.06 | -0.03 – 0.2 |  |  |  |  |  |  |
| G&R-Teacher (Mean) | μ_5_ |  |  |  |  |  |  | 0.6^***^ | 0.06 | 0.5 – 0.7 | 0.5^***^ | 0.06 | 0.4 – 0.7 |
| G&R-Teacher (Deviation) | μ_6_ |  |  |  |  |  |  | 0.5^***^ | 0.05 | 0.4 – 0.6 | 0.5^***^ | 0.04 | 0.4 – 0.6 |
| Age | μ_3_ |  |  |  | 0.21^***^ | 0.03 | 0.15 – 0.3 |  |  |  | 0.2^***^ | 0.02 | 0.1 – 0.2 |
| Male | μ_4_ |  |  |  | -0.06 | 0.14 | -0.3 – 0.2 |  |  |  | 0.06 | 0.11 | -0.2 – 0.3 |
| Number of raters | μ_7_ |  |  |  |  |  |  |  |  |  | 0.2^***^ | 0.06 | -2.1 – -0.9 |
|  | | Random Effects | | |  | | | | | | | | |
| Variance Components |  |  |  |  |  |  |  |  |  |  |  |  |  |
| School | λ_1_ | 0 |  |  | 0.003 | 0.02 |  | 0.02 | 0.03 |  | 0 |  |  |
| Student | λ_2_ | 0.47 | 0.08 | 0.3 – 0.7 | 0.5 | 0.08 | 0.4 – 0.7 | 0.29 | 0.05 | 0.2 – 0.4 | 0.31 | 0.05 | 0.2 – 0.4 |

*Note.* G&R = Grit and Resilience.

^*^*p*<0.05; ^**^*p*<0.01; ^***^*p*<0.001

**Supplementary Table 5**

*Model parameters for mixed-effect models predicting Quality of Life*

|  | Para  meter | Model 1 | | | Model 2 | | | Model 3 | | | Model 4 | | |
| --- | --- | --- | --- | --- | --- | --- | --- | --- | --- | --- | --- | --- | --- |
| Effect |  | Estimate | SE | 95% CI | Estimate | SE | 95% CI | Estimate | SE | 95% CI | Estimate | SE | 95% CI |
|  | | Fixed Effects | | |  | | | | | | | | |
| Intercept | μ_0_ | 0.05 | 0.06 | -0.09 – 0.2 | -0.7^*^ | 0.3 | -1.3 – -0.2 | -0.02 | 0.06 | -0.1 – 0.1 | -1.5^***^ | 0.4 | -2.3 – -0.7 |
| G&R-Parent (Mean) | μ_1_ | 0.5^***^ | 0.07 | 0.4 – 0.7 | 0.5^***^ | 0.06 | 0.4 – 0.6 |  |  |  |  |  |  |
| G&R-Parent (Deviation) | μ_2_ | 0.6^***^ | 0.07 | 0.4 – 0.7 | 0.6^***^ | 0.07 | 0.4 – 0.7 |  |  |  |  |  |  |
| G&R-Teacher (Mean) | μ_5_ |  |  |  |  |  |  | 0.3^***^ | 0.07 | 0.2 – 0.5 | 0.3^***^ | 0.08 | 0.1 – 0.5 |
| G&R-Teacher (Deviation) | μ_6_ |  |  |  |  |  |  | 0.01 | 0.07 | -0.1 – 0.1 | -0.02 | 0.06 | -0.1 – 0.2 |
| Age | μ_3_ |  |  |  | 0.05^*^ | 0.03 | 0.00 – 0.1 |  |  |  | 0.15^***^ | 0.03 | 0.09 – 0.2 |
| Male | μ_4_ |  |  |  | 0.07 | 0.12 | -0.2 – 0.3 |  |  |  | -0.03 | 0.15 | -0.3 – 0.2 |
| Number of raters | μ_7_ |  |  |  |  |  |  |  |  |  | -0.05 | 0.08 | -0.2 – 0.1 |
|  | | Random Effects | | |  | | | | | | | | |
| Variance Components |  |  |  |  |  |  |  |  |  |  |  |  |  |
| School | λ_1_ | 0.004 | 0.02 |  | 0 |  |  | 0 |  |  | 0.003 | 0.02 |  |
| Student | λ_2_ | 0.25 | 0.05 | 0.2 – 0.4 | 0.29 | 0.06 | 0.2 – 0.4 | 0.34 | 0.06 | 0.2 – 0.5 | 0.48 | 0.09 | 0.3 – 0.7 |

*Note.* G&R = Grit and Resilience.

^*^*p*<0.05; ^**^*p*<0.01; ^***^*p*<0.001
